# Precipitation controls the time-lag and cumulative effects of hydrothermal factors on the end of the growing season in a semi-arid region of China

**DOI:** 10.3389/fpls.2024.1483452

**Published:** 2024-11-01

**Authors:** Erhua Liu, Guangsheng Zhou, Xiaomin Lv, Xingyang Song

**Affiliations:** ^1^ State Key Laboratory of Severe Weather, Chinese Academy of Meteorological Sciences, Beijing, China; ^2^ Joint Laboratory of Eco-Meteorology, Chinese Academy of Meteorological Sciences, Zhengzhou University, Zhengzhou, China; ^3^ Collaborative Innovation Center on Forecast Meteorological Disaster Warning and Assessment, Nanjing University of Information Science & Technology, Nanjing, China

**Keywords:** semi-arid region, hydrothermal, phenology, time-lag effect, cumulative effect

## Abstract

Climate change has a substantial influence on the end of the growing season (EOS). The time-lag and cumulative effects are non-negligible phenomena when studying the interactions between climate and vegetation. However, quantification of the temporal effects of climatic factors on the EOS in the context of changing hydrothermal patterns remains scarce. Based on the Moderate Resolution Imaging Spectroradiometer (MODIS) fraction of absorbed photosynthetically active radiation (FPAR), this study first inverted the EOS of typical steppe vegetation in a semi-arid region of China and then quantified the time-lag and cumulative effects of monthly total precipitation (PRE) and monthly average temperature (TEM) on the EOS during 2003–2022. The results showed that a turning point occurred in 2011, when the EOS displayed an advancing trend until 2011, followed by a delayed trend. Accordingly, the climatic background has changed from warming and drying conditions during 2003–2011 to warming and wetting conditions during 2011–2022. The time-lag scales of PRE and TEM on the EOS decreased from 2- and 4-month scales during 2003–2011, respectively, to 1- and 2-month scales during 2011–2022, respectively. The time-lag degree of the hydrothermal factors on the EOS weakened with increased precipitation. The cumulative time scales of the EOS response to PRE and TEM were mainly concentrated within 1-month during different time periods, but the EOS was more sensitive to short-term precipitation. The time lag and cumulative partial correlation coefficient of PRE to EOS changed from mainly negative regulation during 2003–2011 (39.2% and 50.0%, respectively) to mainly positive regulation during 2011–2022 (67.8% and 93.7%, respectively). The time-lag and cumulative effects of TEM on the EOS were positive with the precipitation and temperature gradient under a warming and wetting climate, which indicated that increased precipitation was a prerequisite for temperature to induce a delayed EOS in the semi-arid study region. This study emphasizes the important role of precipitation in regulating the EOS response to hydrothermal factors in semi-arid regions.

## 1 Introduction

Climate variability alters the spatiotemporal patterns of precipitation and temperature, disrupting many ecosystem processes and functions, including carbon and water cycles and energy flows (Keenan et al., 2014), and significantly affects vegetation productivity, biodiversity, and vegetation phenology (Zhu et al., 2016; Chen et al., 2018). Vegetation phenology has drawn widespread research attention due to the close and observable relationships between phenology and climate variables in terrestrial ecosystems (Zhou et al., 2022). The start and end of vegetation phenology are important signals for the inception and cessation of carbon uptake in the ecosystem carbon cycle, respectively, and they play an important role in controlling vegetation productivity as well as water and carbon cycle cycling (Richardson et al., 2010; Wu et al., 2016; Zheng et al., 2020; Zhang et al., 2022a).

In recent decades, global warming has led to the advancement of vegetation green-up and delays at the end of the growing season (EOS) (Tao et al., 2017). However, different directions of phenological change have frequently been reported in the context of current climate warming (Wang et al., 2019a, b; Li et al., 2022). A growing number of studies have focused on the reversal of phenomenon of vegetation phenology (Yang et al., 2014; Li et al., 2018; Liu et al., 2021; Bevacqua et al., 2022; Xiong et al., 2023). For example, in temperate China, the EOS exhibited delays during the 1980s; this trend slowed or even reversed during the 1990s and the 2000s. The green-up in the middle-high latitudes of the Northern Hemisphere showed a gradual reversal from advance to delay. In the Tibetan Plateau region, vegetation green-up showed a similar trend during 1982–2015. To effectively manage and conserve terrestrial biomes, the study of phenological responses and adaptation strategies to climate change is critical for understanding the complex mechanisms of climate–vegetation interactions in ecosystems (Zhang et al., 2019). The phenology of grassland vegetation in arid and semi-arid regions that are sensitive to climate change is influenced mainly by temperature and precipitation (Sha et al., 2016; Tao et al., 2017).

Preseason precipitation and temperature play an important role in regulating vegetation phenology (Guo et al., 2020; Ma et al., 2022; Zhang et al., 2022b). Thus, climate change affects the growth and development of ecosystem vegetation through a complex temporal effect (Mulder et al., 2016; Bigler and Vitasse, 2019). This temporal effect can be divided into the time-lag and cumulative effects. The time-lag effect refers to the effect of climatic factors on vegetation phenology during a certain period before vegetation phenology. The cumulative effect refers to the cumulative effect of climatic factors on vegetation phenology within a certain duration before vegetation phenology. Clarifying the time-lag and cumulative effects of pre-season climate factors (pre-season variables before phenological change begin to the climatic period associated with phenological changes) on phenological change provides important information for understanding climate–vegetation interactions (Guo et al., 2020). The complex interactions between plants and climate variables require further in-depth and systematic analysis (Ding et al., 2020).

Grassland ecosystems are important carbon sinks that are most sensitive to climate change (Hovenden et al., 2008). Grassland vegetation plays a vital role in species conservation (Weber et al., 2018). Therefore, understanding the feedback relationship between climate change and grassland vegetation phenology is crucial to obtain a better understanding of the key processes involved in managing grassland ecosystems. Studies have shown that the contribution of EOS to the trend of growing season length in specific areas is greater than that of green-up (Garonna et al., 2014). However, the response of EOS to climate change is often neglected (Gallinat et al., 2015). The response of EOS to climate change is more complex and variable than that of green-up, which makes it difficult to identify environmental drivers and construct models (Yuan et al., 2020a). Therefore, studying the mechanisms underlying the regulation of vegetation EOS by climate change remains challenging (Yang et al., 2021).

Therefore, quantitative evaluation of the time lag and the cumulative effects of pre-season hydrothermal factors on the EOS in semi-arid regions is critical for improving the performance of phenological models. In this study, it was hypothesized that the time lag and cumulative effects of hydrothermal factors on the EOS would change with changing climatic background. Hydrothermal factors and vegetation types play crucial roles in determining differences in the response of vegetation phenology to climate change (Liu et al., 2021; Ren et al., 2022). Previous studies have focused on the time lag and cumulative effects of climate drivers on the growth and development of different types of vegetation (Ren et al., 2017b; He et al., 2023). However, little is known regarding whether the time lag and cumulative hydrothermal effects on the EOS have changed under ongoing climate change and the underlying mechanisms. Therefore, the main objectives of this study were to (1) quantify the time-lag effect and cumulative effect of the pre-season monthly total precipitation (PRE) and monthly average temperature (TEM) on the EOS of typical steppe vegetation in a semi-arid region of China, and (2) reveal the mechanism of these temporal effects based on PRE and TEM patterns over the past two decades.

## 2 Materials and methods

### 2.1 Study region

**Figure 1:**
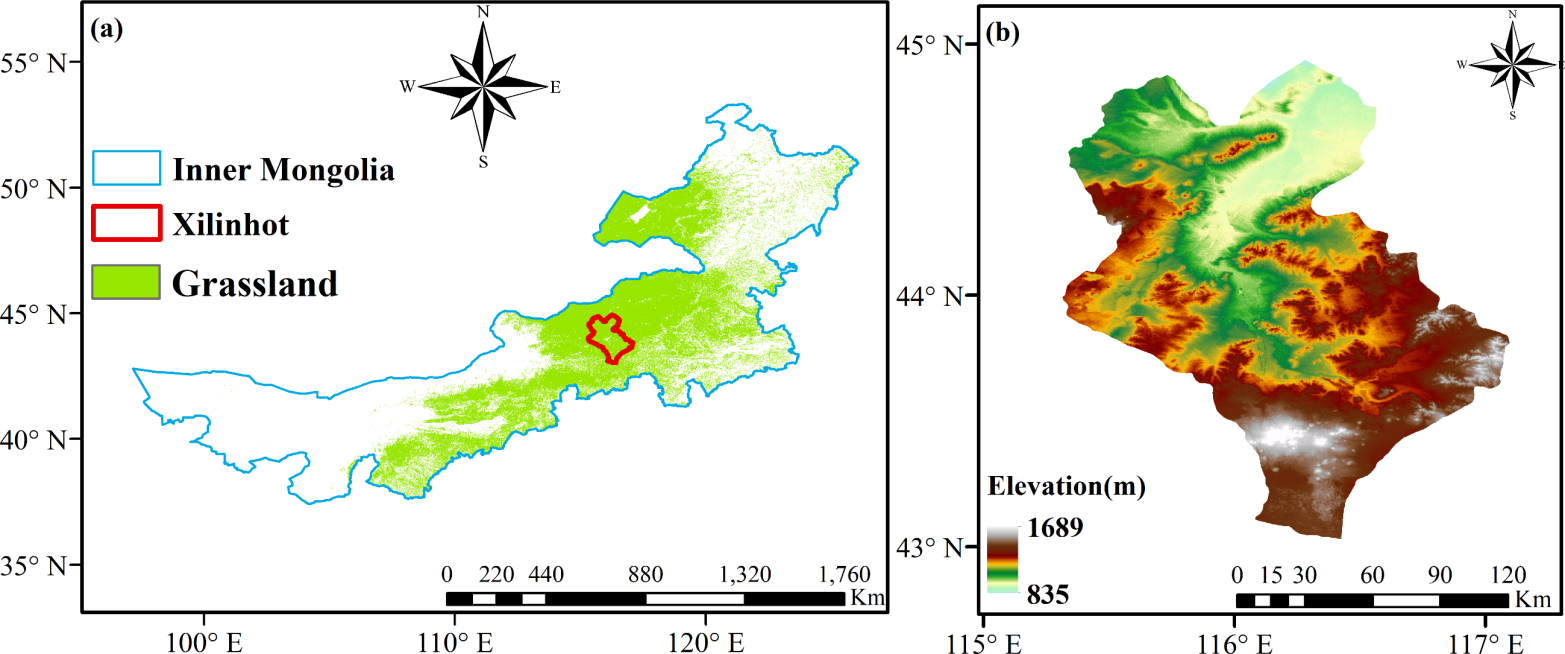
Study region and elevation. **(A)** Geographical location of study region. **(B)** Elevation of study region.

The grassland of Inner Mongolia in northern China is a typical Eurasian steppe region comprising nearly 60% grassland vegetation (Sha et al., 2016). Typical steppe vegetation accounted for 52.8% of the total area. The present study focused on the Xilinhot region of Inner Mongolia (**Figure 1A**), where the vegetation type is dominated by typical steppe vegetation. This area is characterized by a temperate continental monsoon climate, with cold and dry winters and hot and moist summers (Ren et al., 2017b). The elevations in the study area display notable spatial variability, with high elevations in the southeast and low elevations in the north (**Figure 1B**).

### 2.2 Datasets

The Moderate Resolution Imaging Spectroradiometer (MODIS) dataset has been widely used to explore large-scale vegetation phenology. This study utilized the fraction of absorbed photosynthetically active radiation (FPAR) data from MODIS (MCD15A3H.006) for 2003–2022 obtained using the Google Earth Engine (GEE) platform. The FPAR dataset had a spatial resolution of 500 m and temporal resolution of 4 days.

The meteorological data used were obtained from the National Science and Technology Infrastructure Platform National Earth System Science Data Centre (http://www.geodata.cn), including the TEM and PRE during 2003–2022 at a spatial resolution of 0.0083° (approximately 1 km). To ensure consistency in the spatial resolution of the data, monthly climate data were resampled to a spatial resolution of 500 m.

### 2.3 Methods

Using the FPAR dataset, the EOS was inverted using the dynamic threshold method. The inversion process was conducted using TIMESAT 3.3 software. EOS was extracted in two steps. First, a filter function was used to smoothen the FPAR data. Second, the threshold value of the FPAR was defined as 45% to identify the date of EOS.

Partial correlation analysis is a statistical measure of the direction and strength of the linear relationship between the independent variable (*x*) and dependent variable (*y*), which eliminates the interference of one or more covariates (*z =* [*z*_1_ *, z*_2_…, *z*_n_]). The formula used was as follows:


(1) $$R_{x,y|z}=\frac{R_{xy}-R_{xz}*R_{yz}}{\sqrt{\left(1-R_{xz}^{2})*(1-R_{yz}^{2}\right)}}$$

where *R_x,y_*_|_ *_z_* refers to the partial correlation coefficient between *x* and *y* after controlling the variable *z*; *R_xy_*, *R_yz_* and *R_xz_* are the correlation coefficients between *x*, *y*, and *z*.

#### 2.3.1 Time-lag effect

The hydrothermal factors during the early months of the EOS were the most important factors affecting vegetation EOS. In this study, PRE and TEM during the first 6 months of EOS were selected as influencing factors. The time-lag effect refers to the use of 1-month-scale climatic factors, where a time lag of 1 month represents the impact of climatic factors on the EOS during the first month of the EOS, while a time-lag of 6 months represents the impact of climatic factors on the EOS during the sixth month of the EOS. The time-lag effect is determined by the time-lag scales of the EOS, climatic factors, and corresponding maximum partial correlation coefficients. In this study, the time series of the EOS and climatic factors during 2003–2022 (1 ≤ i ≤ 6) were calculated, and the partial correlation coefficients of six time series were calculated (significance level set to *P <*0.05); that is, six partial correlation coefficients were obtained for each pixel for the EOS. The maximum value of the six partial correlation coefficients was considered as the time-lag maximum partial correlation coefficient (*R_max_lagm_*), and the corresponding time-lag scale was recorded. *R_max_lagm_* represents the maximum response of vegetation to the time-lag effect of climatic factors.


(2) $$R_{lagi}=corr(x_{i},y|z_{i}),\;1\leq \text{i}\leq 6$$

(3) $$R_{\max\_lag\text{m}}=\max(|R_{lagi}|)\;1\leq \text{i}\leq 6$$

in the formulas, *i* is the time-lag scale of *i* months. *x_i_* is the time series of climatic factors of *i* months before the EOS. *y* is the EOS time series. *z_i_* is the *z* value of *i* months before the EOS. *R_lagi_* is the time-lag partial correlation coefficient of *y* and *x_i_* after the control variable *z_i_*, and *R_max_lagm_* is the maximum value of *R_lagi_*.

#### 2.3.2 Cumulative effect

To evaluate the degree of response of the EOS to the cumulative effect of climatic factors and their corresponding time scales, a pixel-scale cumulative partial correlation analysis was conducted. First the partial correlation coefficients between the cumulative values of PRE and TEM from the first 1 month to the first 6 months of EOS and EOS were calculated. Subsequently, six groups of partial correlation coefficients were compared, and the maximum pixel values at the corresponding positions were synthesized to obtain the maximum partial correlation coefficient. This indicates the maximum response of the EOS to the cumulative effect of the climate. The corresponding cumulative timescale is the cumulative number of months corresponding to the maximum partial correlation coefficient. The partial correlation coefficients of the six-time series were calculated (based on the data series of the EOS during 2003–2022, with the significance level set at *P <*0.05). The maximum value of the six partial correlation coefficients was recorded as *R_max_cumm_*, and the corresponding cumulative timescale m was recorded.


(4) $$R_{cumi}=corr(x_{i},y|z_{i}),\;1\leq \text{i}\leq 6$$

(5) $$R_{max\_cumm}=\max(|R_{cumi}|)\;1\leq \text{i}\leq 6$$

where *i* is the cumulative time scale; *x_i_* represents the time series of climatic factors with a cumulative time of *i* months; *R_cumi_* is the cumulative partial correlation coefficient of *y* and *x_i_* after the control variable *z_i_*; and *R_max_cumm_* represents the maximum value of *R_cumi_*.

## 3 Results

### 3.1 Spatiotemporal variation patterns of hydrothermal factors and the EOS

**Figure 2:**
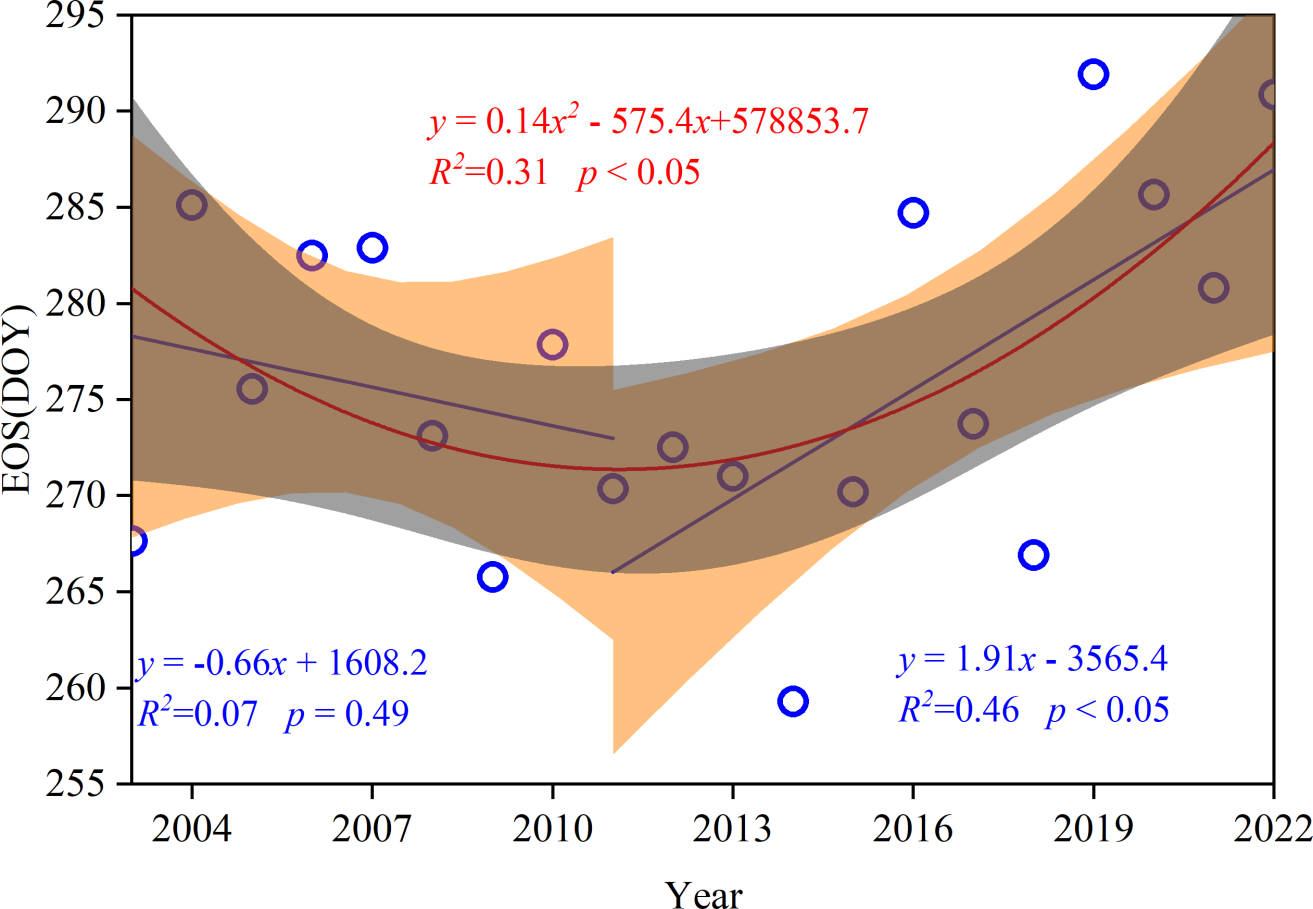
Segmental fitting of the end of the growing season (EOS) time series of typical steppe vegetation in Xilinhot during 2003–2022.

Although the change trend of the EOS was not obvious during 2003–2022, it exhibited phase-change characteristics (**Figure 2**). Based on the EOS rate, the entire study period was divided into two subperiods. The EOS displayed a reversal phenomenon in the semi-arid region, with a slight increase during the first sub-period (2003–2011) and a significant delay during the second sub-period (2011–2022) (*P <*0.05).

**Figure 3:**
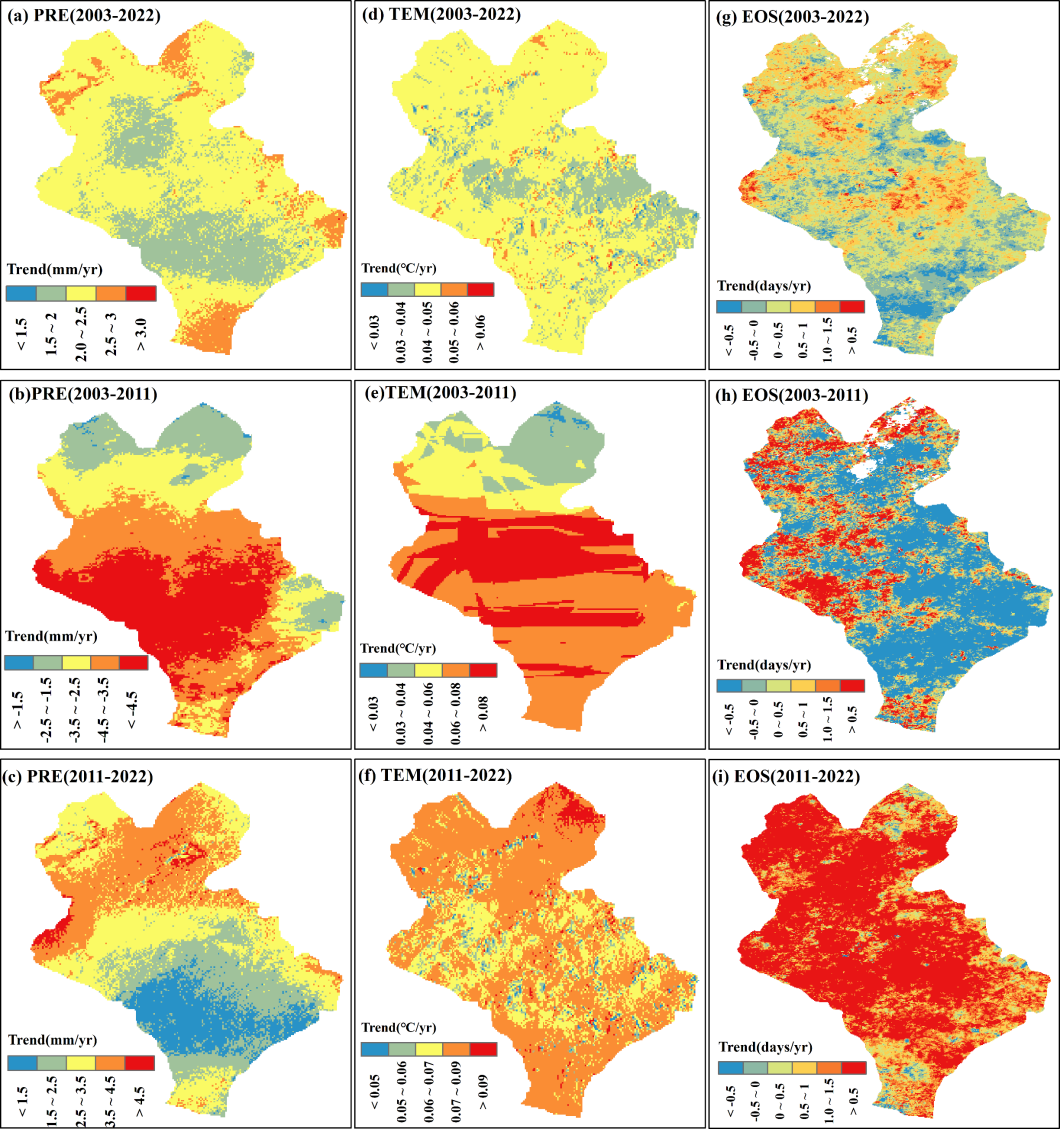
Spatial variation patterns of the monthly total precipitation (PRE), monthly average temperature (TEM), and end of the growing season (EOS) of a typical steppe in the semi-arid region of China during 2003–2022. **(A-C)** Spatial variation trends of the PRE in 2003–2022, 2003–2011, and 2011–2022. **(D-F)** Spatial variation trends of TEM in 2003–2022, 2003–2011, and 2011–2022, respectively. **(G-I)** Spatial variation trends of the EOS in 2003–2022, 2003–2011 and 2011–2022.

The temporal trends of the PRE and TEM during the first 6 months of the EOS and the EOS in the Xilinhot region during 2003–2022 and two subperiods were analyzed (**Figure 3**). The results revealed that the temporal trends and directions of PRE and TEM during different time stages displayed notable spatial variability. Overall, there was an increasing trend in the PRE during 2003–2022 (2.17 mm/yr) (**Figure 3A**). The PRE during 2003–2011 showed a decreasing trend (−3.83 mm/yr) (**Figure 3B**), while the PRE during 2011–2022 exhibited an increasing trend (2.77 mm/yr) (**Figure 3C**). In contrast, the TEM showed an increasing trend during all periods, and the TEM increase rate during 2011–2022 (0.07 °C/yr) was close to that from 2003 to 2011 (0.06°C/yr) (**Figures 3D-F**). This indicates that the spatial and temporal patterns of the PRE and TEM in the study area changed during 2003–2022, and the climate state shifted from a warming and drying climate during 2003–2011 to a warming and wetting climate during 2011–2022. Correspondingly, during 2003–2022, the study found that the temporal trends of the EOS had substantial spatial variability, and the overall trend was slightly delayed (0.27 days/yr) (**Figure 3G**). During the two subperiods, the EOS during 2003–2011 mainly followed a trend of advancing (−0.28 days/yr) (**Figure 3H**), while the EOS showed a delayed trend during 2011–2022 (1.79 days/yr) (**Figure 3I**). This suggests that the direction of change in the EOS has shifted with ongoing climate change.

### 3.2 Time-lag effects of PRE and TEM on the EOS

**Figure 4:**
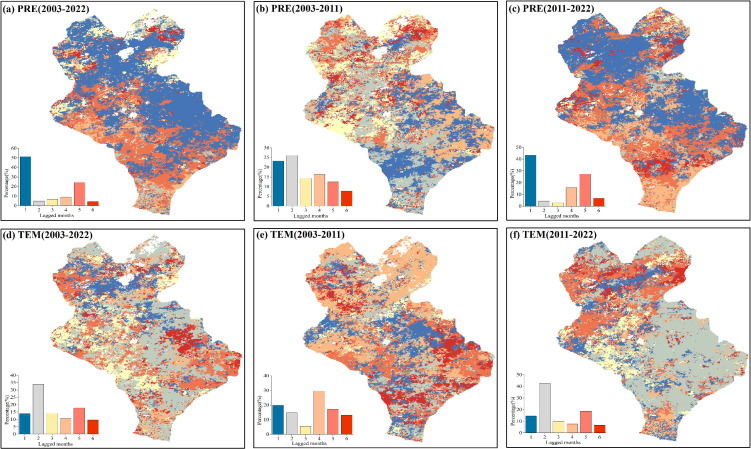
Spatial distributions of the time-lag scales at the end of the growing season EOS response to monthly total precipitation (PRE)and monthly average temperature (TEM). **(A-C)** Spatial distributions of the time-lag scales of the EOS response to PRE in each pixel after controlling for TEM for 2003–2022, 2003–2011, and 2011–2022, respectively. **(D-F)** Spatial distributions of time-lag scales of the EOS response to PRE in each pixel after controlling for PRE during 2003–2022, 2003–2011, and 2011–2022.

The spatial distributions of the time-lag effects of the PRE and TEM on the EOS during different periods were analyzed (**Figure 4**). The time-lag scale of the EOS in response to the PRE in 2003–2022 was dominated by a scale of 1 month, and the proportion of 1-minth pixels was more than 50%. The time-lag scale of the PRE in response to the EOS was dominated by 1- and 2-month from 2003 to 2011, while it was dominated by 1-month during 2011–2022. This suggests that the time-lag degree of the EOS in response to the PRE was stronger under warming and drying climate conditions. The time-lag scale of the EOS in response to TEM during 2003–2022 was dominated by the 2-month scale, which accounted for 35% of the total pixels. In particular, the time-lag scale of the response of the EOS to TEM was dominated by the 4-month scale during 2003–2011, while it was dominated by the 2-month scale during 2011–2022, which indicated that the time-lag degree of the EOS in response to TEM was also stronger under the warming and drying climate conditions. Taken together, the time-lag scales of the influence of climatic factors on EOS decreased with an increase in PRE. The time-lag scales of the influence of TEM on the EOS lasted longer, whereas the EOS was mainly influenced by short-term precipitation.

**Figure 5:**
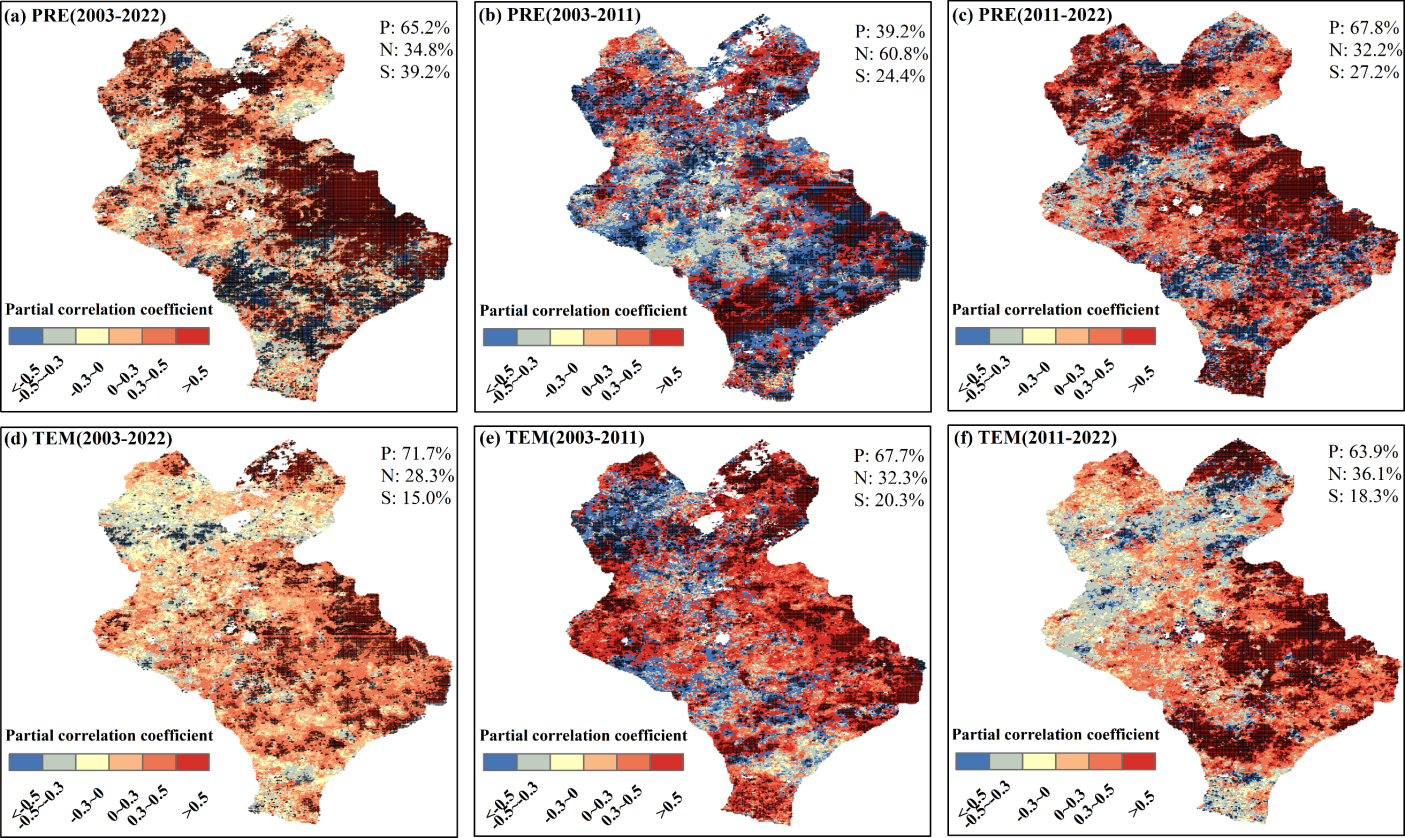
Spatial distributions of the time-lag maximum partial correlation coefficient between the end of the growing season (EOS), monthly total precipitation (PRE), and monthly average temperature (TEM). **(A-C)** Spatial distributions of the time-lag maximum partial correlation coefficient between the EOS and PRE after controlling for TEM for 2003–2022, 2003–2011, and 2011–2022, respectively. **(D-F)** Spatial distributions of the time-lag maximum partial correlation coefficient between the EOS and TEM after controlling for PRE for 2003–2022, 2003–2011, and 2011–2022, respectively. + denotes significant correlation. P, N, and S denote the positive, negative, and significant correlations, respectively.

**Figure 6:**
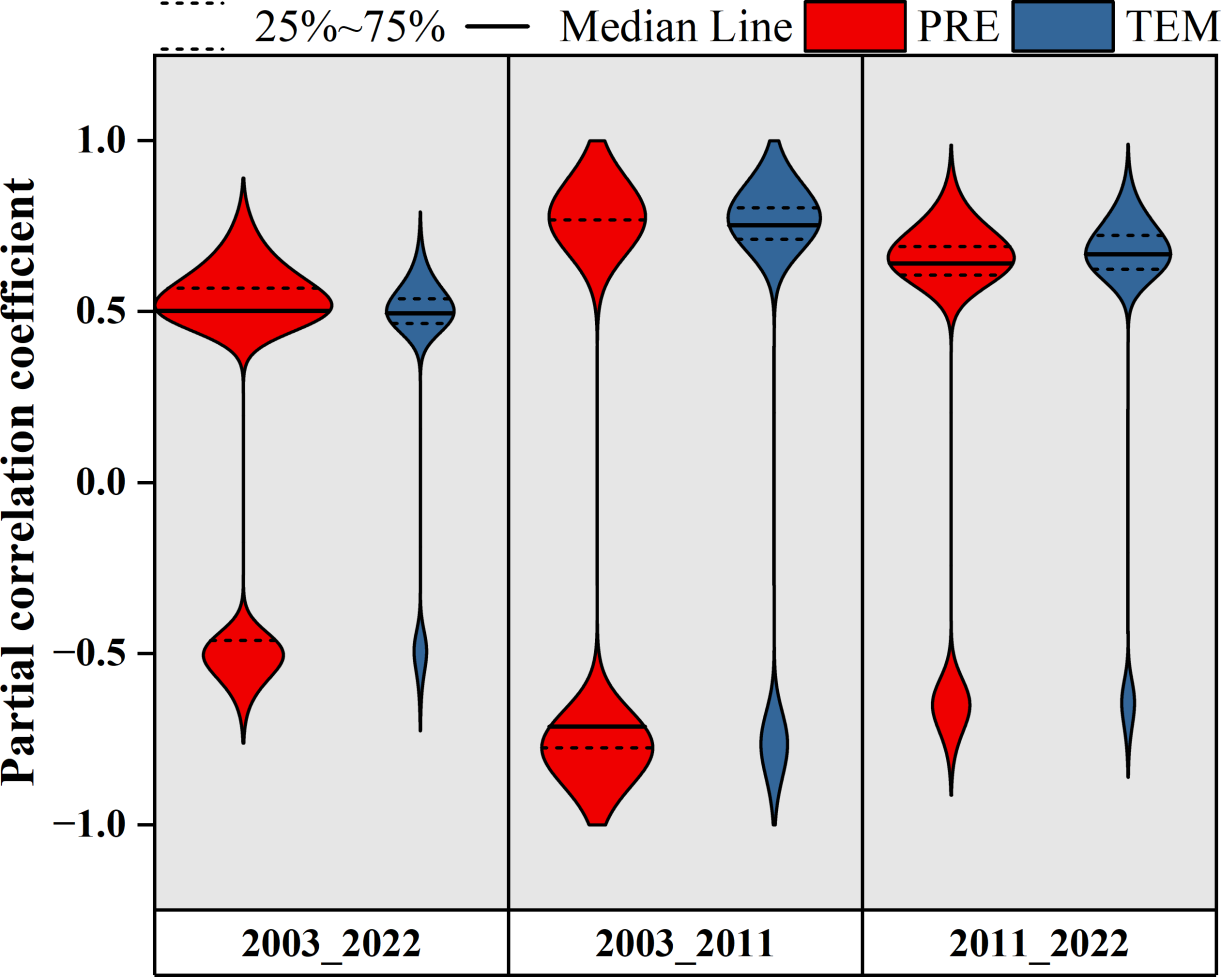
Violin plot of the time-lag maximum partial correlation coefficients between the end of the growing season (EOS), monthly total precipitation (PRE), and monthly average temperature (TEM). The width of each violin plot represents the probability density of partial correlation coefficients at different values. The figure is related to **Figure 5**, which shows only the significant pixels (*P <*0.05).

The spatial distributions of the time-lag maximum partial correlation coefficients of different pixels demonstrated that the time-lag effects of PRE and TEM on the EOS during 2003–2022 were mainly positive, and the proportions of pixels were 65.2% and 71.7%, respectively (**Figures 5A, D**). Among them, the proportion of significant pixels for the time-lag effect of PRE on EOS (39.2%, *P <*0.05) was higher than that for TEM (15.0%, *P <*0.05) (**Figure 6**). Significant pixels were mainly distributed in the eastern part of Xilinhot. The time-lag effect of PRE on the EOS was mainly negative (60.8%) during 2003–2011, whereas the time-lag effect of TEM on the EOS was mainly positive during the same period (67.7%) (**Figures 5B, E**, **6**). During 2011–2022, the time-lag effects of PRE and TEM on the EOS were mainly positive, and the proportions of the pixels were 67.8% and 63.9%, respectively (**Figures 5C, F**). The proportion of significant pixels for the time-lag effect of PRE on EOS (27.2%, *P <*0.05) was higher than that for TEM (18.3%, *P <*0.05) (**Figure 6**).

**Figure 7:**
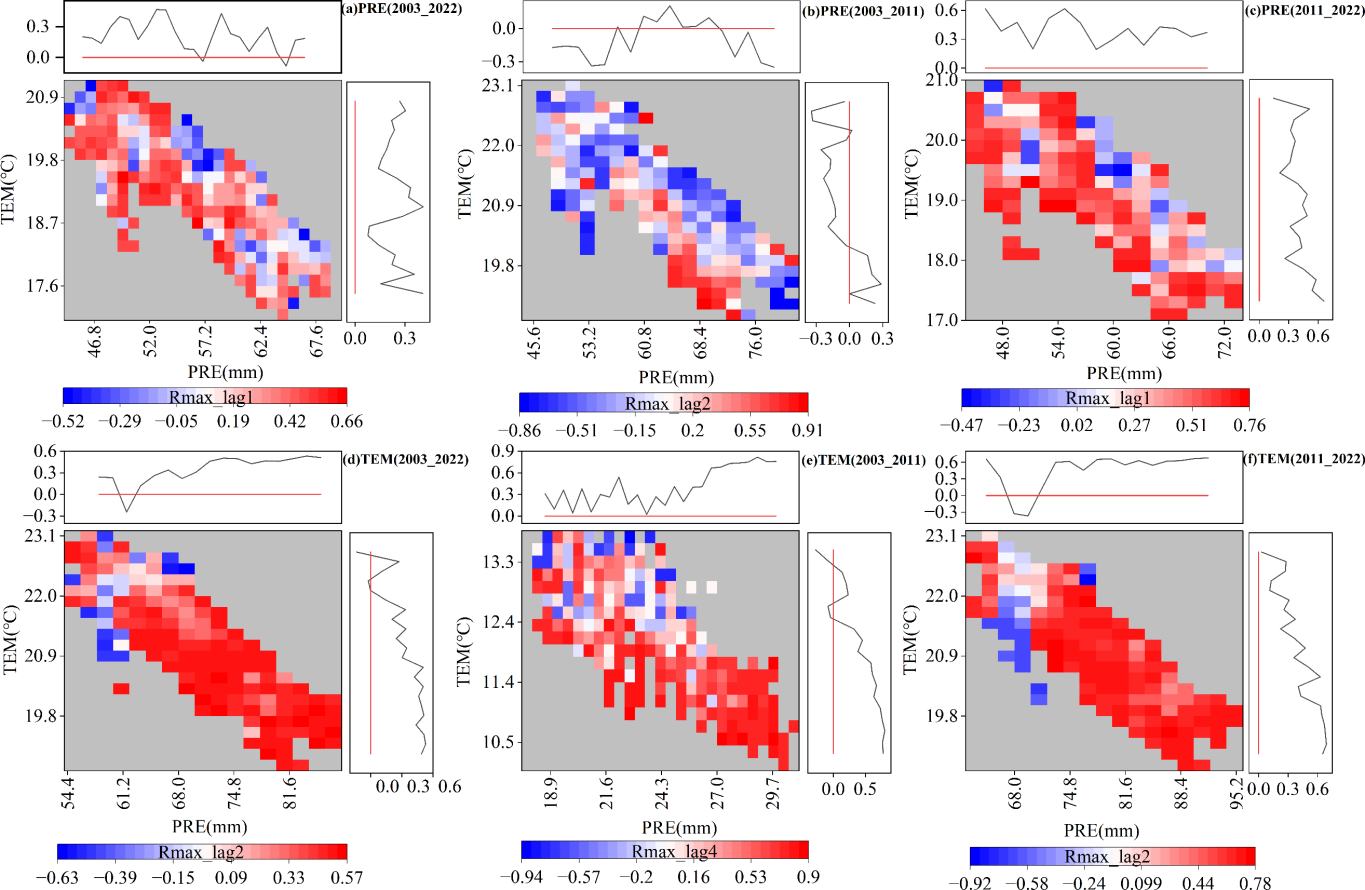
Distributions of time-lag maximum partial correlation coefficients with the gradient of monthly average temperature (TEM) and monthly total precipitation (PRE). **(A-C)** Partial correlation coefficients between PRE and end of the growing season (EOS) for 2003–2022, 2003–2011, and 2011–2022, respectively. **(D-F)** Partial correlation coefficients between TEM and EOS for 2003–2022, 2003–2011, and 2011–2022. The figure is related to **Figure 5**, showing only significant pixels (*P <*0.05).

Based on the above analysis, the distributions of the time-lag maximum partial correlation coefficients under PRE and TEM gradients were further explored (**Figure 7**). The PRE partial correlation coefficients were greater than zero for both the entire study period of 2003–2022 and the subperiod of 2011–2022, suggesting that the increase in PRE and TEM was conducive to the postponement of the EOS under the current TEM and PRE gradients (**Figures 7A-C**). In contrast to the above two stages, during the subperiod 2003–2011, the variations in the partial correlation coefficient of PRE and TEM were less than zero, which indicated that under the climate background during this stage, the increase in PRE and TEM was not conducive to the postponement of the EOS. The TEM partial correlation coefficients were greater than zero in the climatic context of all three phases (**Figures 7D-F**). Moreover, the TEM partial correlation coefficient decreased with increasing TEM and increased with increasing PRE, indicating that excessive TEM was not conducive to the delay or advancement of the EOS. In general, the results showed that the time-lag effect of climatic factors on the EOS was related to the climatic background. Sufficient precipitation is a prerequisite for temperature to delay EOS.

### 3.3 Cumulative effects of PRE and TEM on the EOS

**Figure 8:**
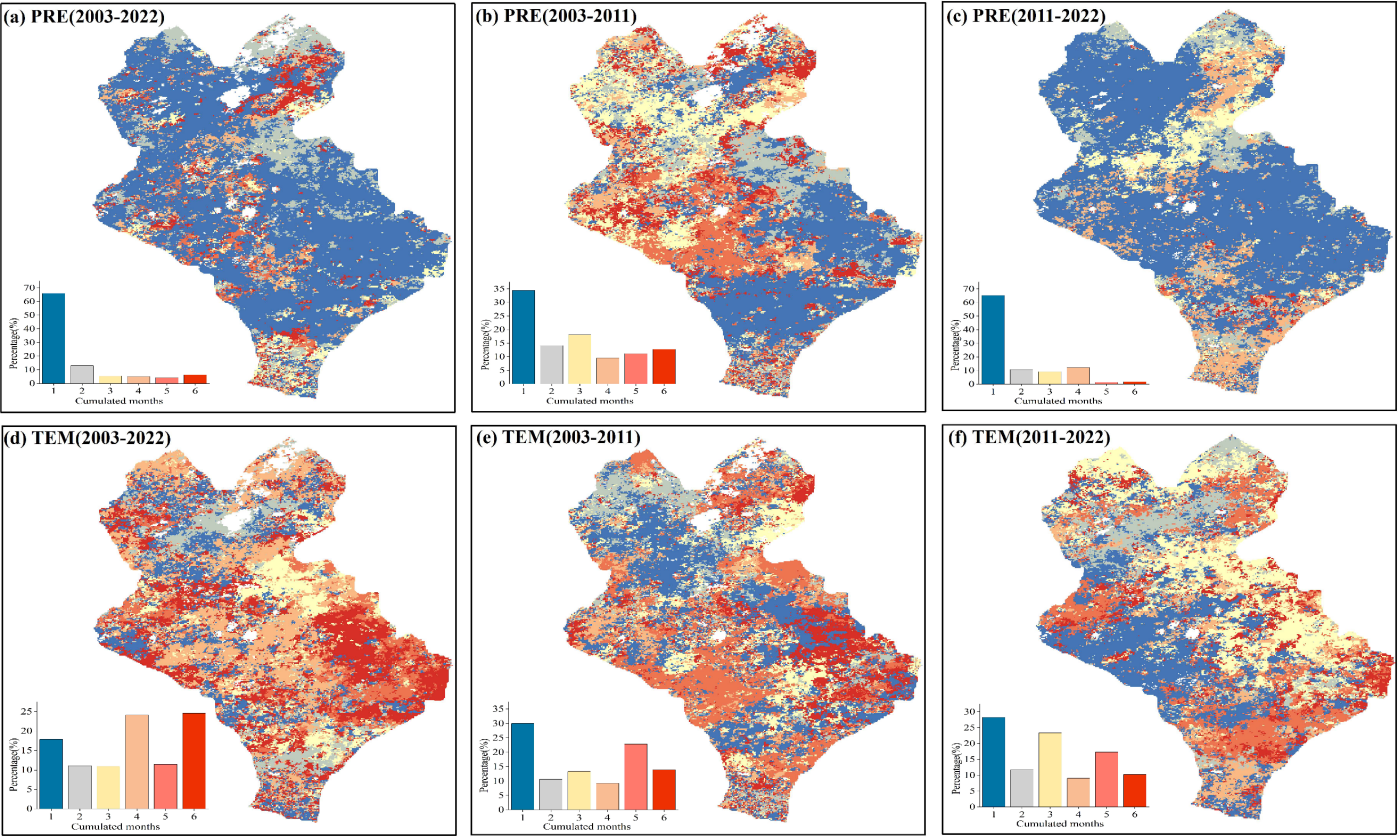
Spatial distributions of the cumulative time scales of the response at the end of the growing season (EOS) to monthly total precipitation (PRE) and monthly average temperature (TEM). **(A-C)** Spatial distributions of the cumulative time scales of the impact of PRE on the EOS after controlling for TEM for 2003–2022, 2003–2011, and 2011–2022, respectively. **(D-F)** Spatial distributions of the cumulative time scales of the impact of TEM on EOS after controlling for PRE for 2003–2022, 2003–2011, and 2011–2022, respectively.

The spatial distributions of the cumulative time scales of the EOS response to PRE and TEM were analyzed (**Figure 8**). For PRE (**Figures 8A-C**), the cumulative time scales of the response of the EOS to PRE in the three stages mainly consisted of the 1-month scale. The proportion of pixels with a 1-month scale exceeded 60% for both 2003–2022 and 2011–2022, whereas it was below 35% during 2003–2011. This indicated that the cumulative effect of PRE on EOS was enhanced under a warming and drying climate. For TEM (**Figures 8D-F**), the cumulative time scales of the EOS response to TEM during 2003–2022 mainly consisted of 4- and 6-month scales, which accounted for 50% of the pixels. In terms of different stages, the cumulative time scale of the EOS response to TEM during 2011–2022 primarily consisted of the 1-month, while the proportion of pixels with a cumulative time scale greater than 1 month was significantly increased. This demonstrates that the cumulative effect of TEM on EOS was stronger than that of PRE.

**Figure 9:**
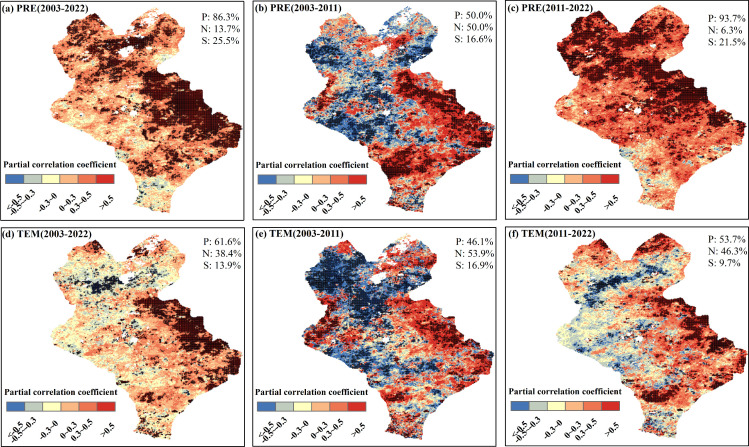
Spatial distributions of the cumulative maximum partial correlation coefficients between the end of the growing season (EOS) and monthly total precipitation (PRE) and monthly average temperature (TEM). **(A-C)** Spatial distributions of the cumulative maximum partial correlation coefficients between EOS and PRE after controlling for TEM for 2003–2022, 2003–2011, and 2011–2022, respectively. **(D-F)** Spatial distributions of the cumulative maximum partial correlation coefficients between the EOS and TEM after controlling for PRE for 2003–2022, 2003–2011, and 2011–2022, respectively. + denotes significant correlation. P, N, and S denote the positive, negative, and significant correlations, respectively.

**Figure 10:**
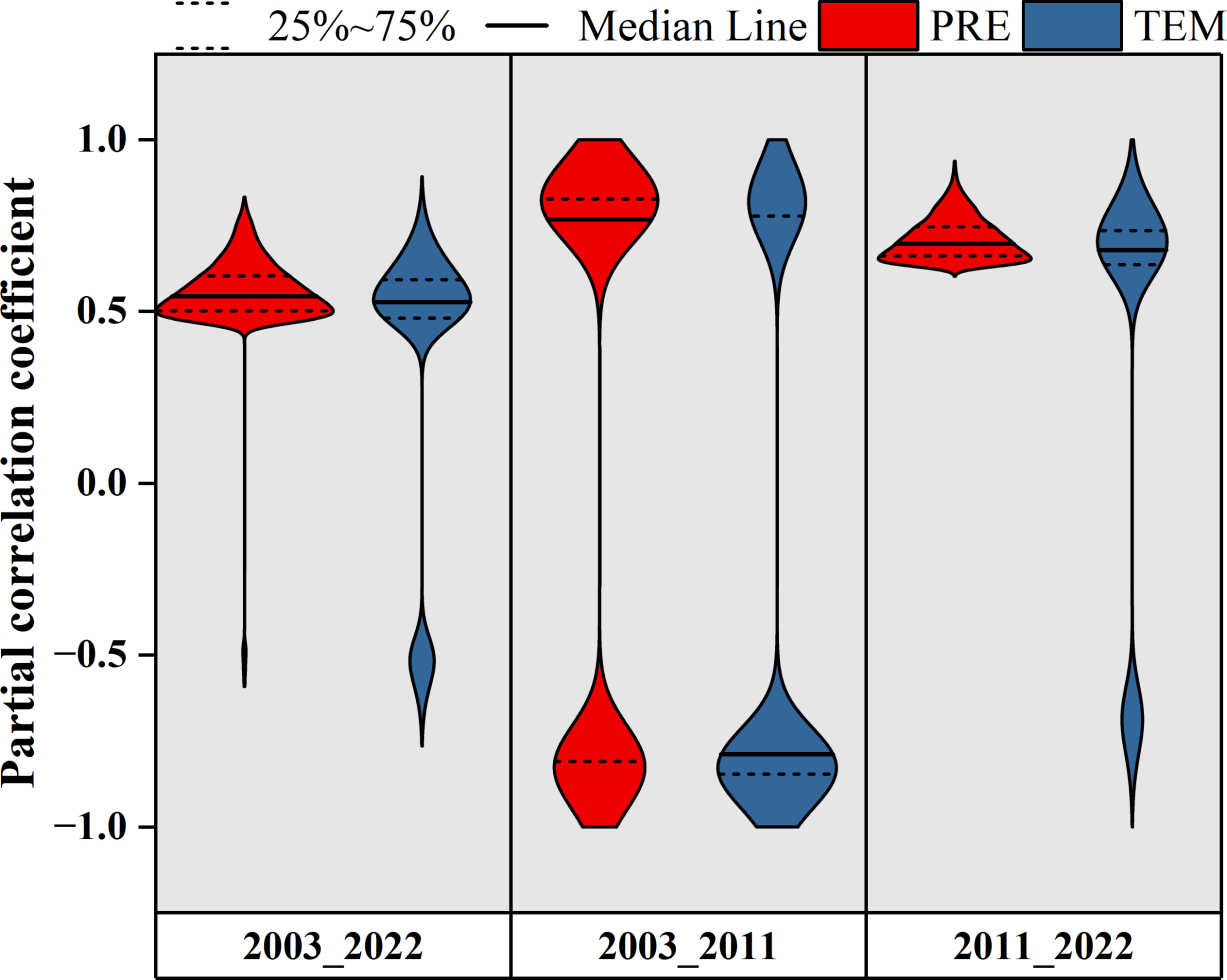
Violin plot of the cumulative maximum partial correlation coefficients between the end of the growing season (EOS), monthly total precipitation (PRE), and monthly average temperature (TEM). The width of each violin plot represents the probability density of the partial correlation coefficients at different values. The figure is related to **Figure 9**, which shows only the significant pixels (*P <*0.05).

The spatial distributions of the maximum cumulative partial correlation coefficients of the pixels are shown in **Figure 9**. The cumulative effects of PRE and TEM on EOS during 2003–2022 were mainly positive, and the proportion of pixels was 86.3% and 61.6%, respectively (**Figures 9A, D**). Among them, the proportion of significant pixels for the cumulative effect of PRE on EOS (25.5%, *P <*0.05) was higher than that of TEM (13.9%, *P <*0.05) (**Figures 9A, D**, **10**). This scenario was particularly apparent in the eastern part of Xilinhot. The proportions of pixels with positive and negative cumulative effects of PRE on the EOS were similar during the 2003–2011 period, while the cumulative effect of TEM on the EOS was mainly negative (**Figures 9B, E**, **10**). The cumulative effects of PRE and TEM on EOS were mainly positive during the 2011–2022 period, and the proportions of pixels were 93.7% and 53.7%, respectively (**Figures 9C, F**). The proportion of significant pixels for the cumulative effect of PRE (21.5%, *P <*0.05) on EOS was higher than that of TEM (9.7%, *P <*0.05), and the cumulative effect of PRE was positive (**Figure 10**). In general, the cumulative effects of the PRE and TEM on the EOS were altered by the precipitation regime. The influence of PRE on EOS changed from negative regulation to positive regulation over time.

**Figure 11:**
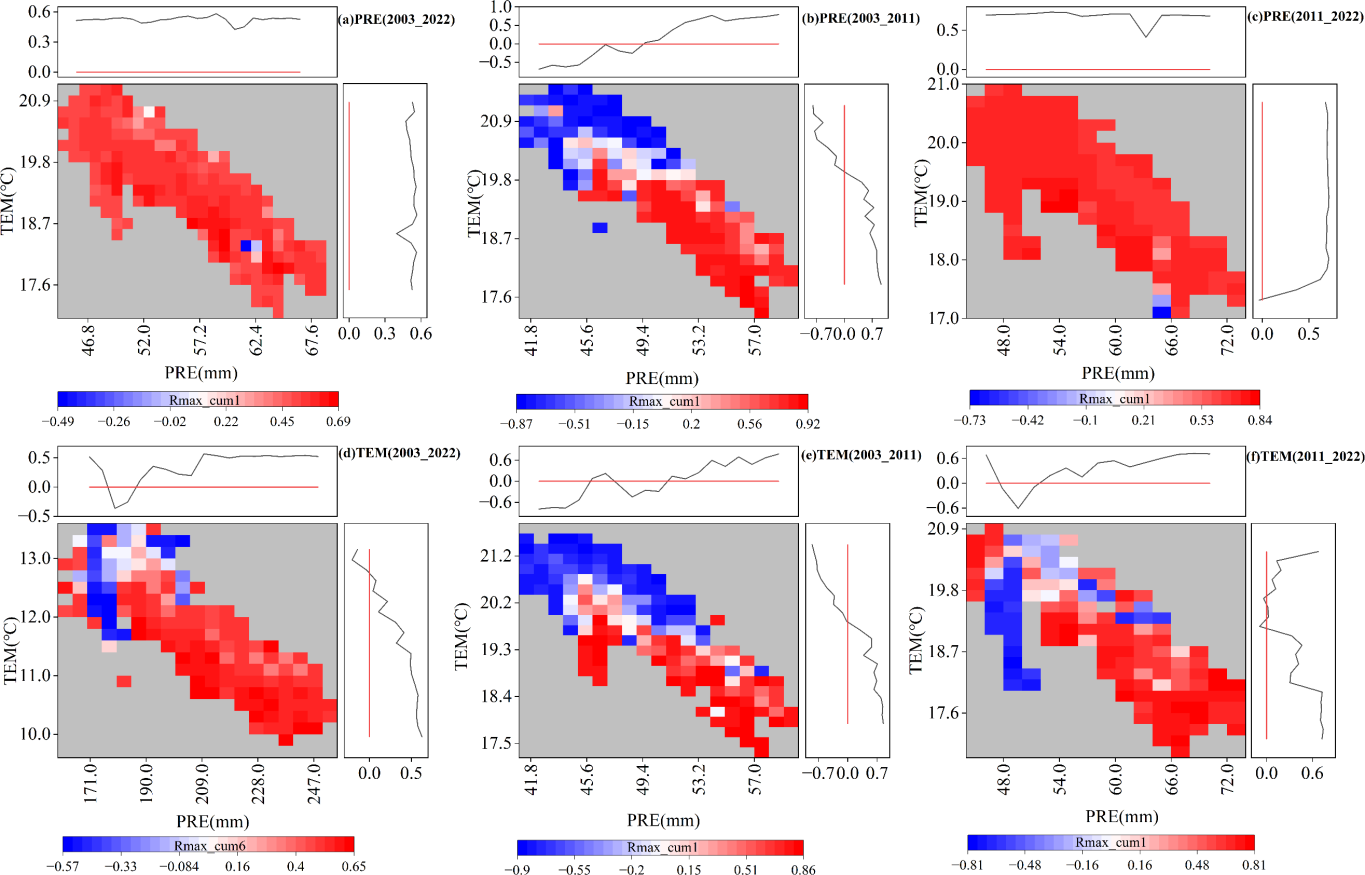
Distributions of the cumulative maximum partial correlation coefficients with the gradient of monthly average temperature (TEM) and monthly total precipitation (PRE). **(A-C)** Partial correlation coefficients between PRE and the end of the growing season (EOS) for 2003–2022, 2003–2011, and 2011–2022, respectively. **(D-F)** Partial correlation coefficients between TEM and EOS for 2003–2022, 2003–2011, and 2011–2022. The figure is related to **Figure 9**, showing only significant pixels (*P <*0.05).

Based on the above analysis, the distributions of the cumulative maximum partial correlation coefficients under PRE and TEM gradients were further explored (**Figure 11**). The cumulative partial correlation coefficient of PRE (**Figures 11A-C**) was greater than zero during the entire study period of 2003–2022 and the sub-period of 2011–2022, which indicated that under the current TEM and PRE gradient, increased PRE and TEM values were conducive to the delay of the EOS. In contrast to the above two stages, the partial correlation coefficient of PRE in 2003–2011 shifted from negative to positive with an increase in PRE, and from positive to negative with an increase in TEM, which indicated that under the climate background during this stage, increased PRE before the EOS was conducive to the delay of the EOS to a certain extent, while increased TEM was not conducive to the delay of the EOS. The partial correlation coefficient of TEM (**Figures 11D-F**) changed from negative to positive with the increase in PRE during 2003–2011 and from positive to negative with the increase in TEM, which was in accordance with the change rule of the PRE partial correlation coefficient. The partial correlation coefficient of TEM during 2011–2022 was greater than zero, demonstrating that, under the current climate background, increased PRE and TEM values were conducive to the delay of the EOS. In general, the increase in TEM under the climate background of increased PRE was conducive to the postponement of EOS.

## 4 Discussion

The EOS did not change significantly over the period of 2003–2022 for the entire study area. The temporal trends of the EOS exhibited spatial variability, and the trend in the EOS changed from an advance to a delay. Therefore, the reversal of the EOS may be closely related to climate warming and the precipitation regime shift. The response of vegetation phenology to pre-season precipitation and temperature exhibits spatial variability in terms of its intensity and direction (Zhang et al., 2022b), which is primarily caused by differences in the spatial distribution patterns of precipitation and temperature (Fu et al., 2020; Ren et al., 2022). Studies have demonstrated that spatial and temporal patterns of precipitation and temperature are key factors controlling the spatial differentiation of grassland vegetation phenology (Ren et al., 2017a). In addition to climate warming, changes in precipitation patterns constitute another aspect of climate change (Fu et al., 2020). Previous reports have shown that changes in precipitation are a more important factor than temperature in driving shifts in vegetation phenology in semi-arid regions (Ren and Peichl, 2021; Currier and Sala, 2022). Pre-season cumulative precipitation has the greatest positive impact on vegetation in arid and semi-arid regions (Guo et al., 2021). The results of the current study provide evidence of the dynamic nature of climatic constraints on the EOS and show that the influence of precipitation on the EOS has changed from mainly negative regulation to mainly positive regulation. These findings may provide an approach to improve model performance by considering shifting dominant factors under changing climatic conditions (Fu et al., 2020).

The present study found that the pre-season PRE and TEM were mainly positively correlated with the EOS of typical steppe vegetation under warming and wetting climate conditions, which is consistent with previous findings (Liu et al., 2016; Zhang et al., 2020). This may be attributed to the fact that higher temperatures enhance the activity of photosynthetic enzymes and slow the degradation of chlorophyll, thus leading to a delay in EOS (Richardson et al., 2013; Wu et al., 2018). However, a previous study found that the EOS of herbaceous plants in the Qinghai–Tibet Plateau advanced with an increase in precipitation and was more sensitive to temperature, which was contrary to the results of the present study (Zhu et al., 2017). This discrepancy may be attributed to the relatively abundant precipitation and extremely cold temperatures in high-altitude areas, where temperature is the limiting factor for alpine grassland growth. In contrast, in the arid southwestern part of the Tibetan Plateau, increased pre-season precipitation was found to delay EOS (Shen et al., 2023). This implies that the climate–phenology relationship varies greatly in different regions and vegetation types (Cong et al., 2012; Zhang et al., 2022b) and that the response of vegetation phenology to climate change is complex and changeable (Ganjurjav et al., 2016; Yuan et al., 2020b; Bevacqua et al., 2022). Thus, the present results suggest that a delayed trend in the EOS of typical grasslands is likely if precipitation thresholds are reached earlier. Therefore, as climatic conditions under climate change shift from warming and drying conditions to warming and wetting conditions, the precipitation and temperature thresholds that trigger the inversion of the EOS deserve further study.

In this study, both PRE and TEM had a strong time lag and cumulative effect on the EOS of typical steppe vegetation in the semi-arid study region. The time lag and cumulative time scales were mainly concentrated at the pre-season 1-month scale, indicating that the EOS of typical steppe vegetation in the semi-arid region responded quickly to hydrothermal factors. A previous study showed that the climatic factors 1 month before the EOS were most closely related to the EOS on the Qinghai–Tibet Plateau (Liu et al., 2021), which was consistent with the results of the present study. In addition, the proportion of pixels with time lag and cumulative effects of TEM on the EOS above the 1-month time scale was greater than that for PRE, further implying that the EOS responded more strongly to short-term precipitation than to temperature, which was inconsistent with a previous report (He et al., 2023). The present study demonstrated that with increasing precipitation, the time-lag scale of PRE and TEM on the EOS was shortened, indicating that increased PRE weakened the time-lag effect of hydrothermal factors on the EOS. In addition, with an increase in the PRE, the response of the EOS to TEM gradually increased. A previous study also found that the temperature sensitivity of the EOS in temperate steppe increased with an increase in precipitation (Yang et al., 2014). Research has shown that, under sufficient water and nutrient conditions, temperature is the dominant factor in delaying the EOS of vegetation (Fu et al., 2018b). In other words, temperature triggers changes in vegetation phenology only when water requirements are met (Ren et al., 2022). Therefore, water availability regulates the sensitivity of EOS to temperature. The novelty of the present study lies in the observation that the regulatory effects of PRE and TEM on EOS change with changing climatic conditions.

The time lag and cumulative effects of PRE and TEM on the EOS of typical steppe grasslands in the semi-arid region of China demonstrated that pre-season climatic factors have a strongly influenced on the EOS. This study provides a unique perspective for assessing the PRE and TEM control of phenology, and believed the findings imply that climatic context plays a key role in the EOS in response of the EOS to PRE and TEM. The intensity and direction of the influence of climate driving factors on EOS exhibited spatiotemporal differences (Yuan et al., 2020a). In areas with insufficient precipitation, an increase in temperature leads to enhanced the increase of surface evaporation, which may be the reason for the advancement of the EOS in these areas (Fu et al., 2018a). In contrast, increased precipitation can improve the hydrothermal conditions and delay the EOS (Ma et al., 2023). Warming or drying of the climate may change alter the phenological patterns and species composition of ecosystems (Zhu et al., 2016). The present study found that different climatic backgrounds led to inconsistent time lags and cumulative effects in the EOS response of the EOS to hydrothermal factors. Under the warming and drying conditions, the partial correlation coefficient of the PRE with the gradient of the TEM and PRE was less than zero, indicating that the increased of PRE in the climatic background was not conducive to the delay of the EOS under these conditions. This resultat may be due to the climatic background of the less lower moisture availability in the summer (July), and the increased PRE enhanced increase of soil moisture, thereby promoting vegetation to promote photosynthesis of vegetation, which prompted the EOS to advance. This mechanism is an adaptive measure for plants to cope with climate change. The time-lag partial correlation coefficients of TEM were greater than zero during 2003–2011 and decreased and increased with increasing TEM and PRE, respectively. In other words, as the TEM gradually increased TEM, the tendency of TEM to delay the EOS was weakened, which may be related to the drought stress caused by warming (He et al., 2021), further negatively affecting impacting vegetation productivity (Sharma et al., 2016). The time-lag partial correlation coefficients of PRE and TEM were greater than zero during 2011–2022, which indicated that under the warming and wetting climate, increases in PRE and TEM were conducive to the delay of the EOS. This finding provides further evidence that increased precipitation, rather than warming, significantly delays EOS in the semi-arid study region.

## 5 Conclusion

This study examined the spatial and temporal characteristics of the time lag and cumulative effects of PRE and TEM on EOS in the semi-arid region during 2003–2022. The results reflect the important role of the climate background in regulating the response of the EOS to TEM and PRE. The time-lag scales of the EOS response to PRE and TEM were shortened as the climate shifted from warm and dry conditions to warm and wet conditions. Moreover, the time-lag effect of PRE on the EOS changed from negative (60.8%) for 2003–2011 to positive (67.8%) during 2011–2022, indicating that the time-lag effect of hydrothermal factors on the EOS changed with climate background. The cumulative time scale of the PRE to the EOS was concentrated within the 1-month (65%) scale during 2011–2022. The proportion of pixels with cumulative time scales for TEM to the EOS of greater than 1 month was greater than that for PRE during the subperiod of 2011–2022, indicating that the EOS was highly sensitive to short-term precipitation. Under a warming and drying background during 2003–2011, the cumulative partial correlation coefficients of PRE and TEM changed from positive to negative with a decrease in PRE and an increase in TEM, respectively. In addition, the increase in TEM was conducive to the postponement of the EOS under a warming and wetting background during 2011–2022. The change in the primary drivers of the EOS was chiefly attributed to significant increases in pre-season precipitation. Increased precipitation is a prerequisite for increasing temperature to delay EOS. These results provide an important reference for the construction of vegetation phenology models.

## Data Availability

Publicly available datasets were analyzed in this study. This data can be found here: http://www.geodata.cn.
